# Thin Film Deposition by Atmospheric Pressure Dielectric Barrier Discharges Containing Eugenol: Discharge and Coating Characterizations

**DOI:** 10.3390/polym12112692

**Published:** 2020-11-16

**Authors:** Tsegaye Gashaw Getnet, Milton E. Kayama, Elidiane C. Rangel, Nilson C. Cruz

**Affiliations:** 1 Department of Chemistry, College of Science, Bahir Dar University, Bahir Dar 79, Ethiopia; 2 Laboratory of Technological Plasmas, Institute of Science and Technology, Sao Paulo State University, Sorocaba, SP 18087-180, Brazil; 3 Laboratory of Plasma and Applications, Sao Paulo State University, Campus at Guaratinguetá, Guaratinguetá, SP 12516-410, Brazil; milton.kayama@unesp.br (M.E.K.); elidiane.rangel@unesp.br (E.C.R.)

**Keywords:** Eugenol, plasma, dielectric barrier discharge

## Abstract

Eugenol (4-Allyl-2-methoxyphenol) is the main constituent of clove oil. In addition to being widely used as a condiment, it has been recognized as a powerful bactericide. Owing to that, Eugenol has been used in several applications including odontology and as a conservative for food products. Aiming at the development of natural bactericide coatings, in this work, using an atmospheric pressure plasma in a dielectric barrier discharge (DBD) reactor Eugenol was deposited on stainless steel substrate, with argon as a carrier gas. The discharge power supply was a transformer at 14.4 kV peak-to-peak voltage and 60 Hz frequency. Operating with a gas flow rate at 4 L/min, the active power was around 1.2 W. The maximum plasma electron temperature of the plasma with monomers was about 1.5 eV, estimated by visible emission spectroscopy using a local thermodynamic equilibrium approach. The study also comprehended the analysis of the film structure, aging, and thermal stability using infrared reflectance spectroscopy, and its thicknesses and roughness by profilometry. The thickness of the films was in the range of 1000 to 2400 nm with a roughness of up to 800 nm with good adhesion on the substrate. The FTIR result shows a stable coating with a chemical structure similar to that of the monomer. Aging analysis showed that the film does not degrade, even after exposing the film for 120 days in ambient air and for 1.0 h under a high thermal UV-lamp.

## 1. Introduction

Atmospheric dielectric barrier discharge (DBD) plasmas have drawn much attention due to low experimental cost, easy handling, and having no need for expensive vacuum systems [1,2,3]. DBD plasmas are the best choice for a variety of industrial applications, including large scale ozone generation, odor removal, and material processing [4,5]. In addition, the potential of non-thermal DBD plasma systems for various medical and biological applications including wound healing, tissue regeneration, blood coagulation, tooth bleaching, and cancer treatment has been demonstrated [6,7,8,9,10,11,12,13,14,15,16,17,18]. In the midst of such applications, thin-film deposition using DBD plasmas presents several benefits compared to its vacuum counterparts in a variety of applications, such as film deposition on vacuum- or temperature-sensitive substrates and on biological/living surfaces [19,20]. The deposition by DBD is able to tailor the physical and chemical properties of different substrates to a specific application through the modification of the deposition parameters, such as applied voltage, type, and flux of the monomer, and deposition time [21].

Employing plasmas, it is possible to deposit films with adjustable properties on a variety of substrates, in principle, using any organic vapor, including even those non-polymerizable via conventional pathways [22,23,24] such as film precursors. For instance, the successful deposition of films using low-pressure plasmas containing natural extracts, such as eucalyptus and lavender oils [25], terpene-4-ol [23], and linalyl acetate [26] has been reported. It is interesting to point out that a wide range of applications of those films in electronics, biomaterials, nanotechnology, and protective coatings has been investigated [27,28,29]. In this context, Eugenol (Eu), an extract obtained from clove, is particularly interesting. Used as a condiment since ancient times, it also presents intense antimicrobial activity against free-living microorganisms. Therefore, it can be considered a very promising material to be used in the production of bactericidal coatings for food packing and other related products. However, to be of practical interest such coatings must be uniform, defect-free, and well adhered to different types of substrates. As well documented in the literature, such requirements can be perfectly met using plasma-based deposition techniques. Although several experimental studies have been conducted addressing the fundamental mechanisms and chemical phenomena of atmospheric DBD plasmas [30,31,32], there is not, as far as we know, any detailed study on the deposition of films from essential oils using DBD plasmas. In this regard, plasma diagnostic techniques such as electrical measurements and optical emission spectroscopy can be useful tools to help in understanding the underlying physical and chemical processes [31,32,33,34,35]. In this context, the present study investigated the film deposition from atmospheric DBD plasmas containing Eugenol vapor. Both plasmas and coatings properties were characterized.

## 2. Materials and Methods

**Figure 1:**
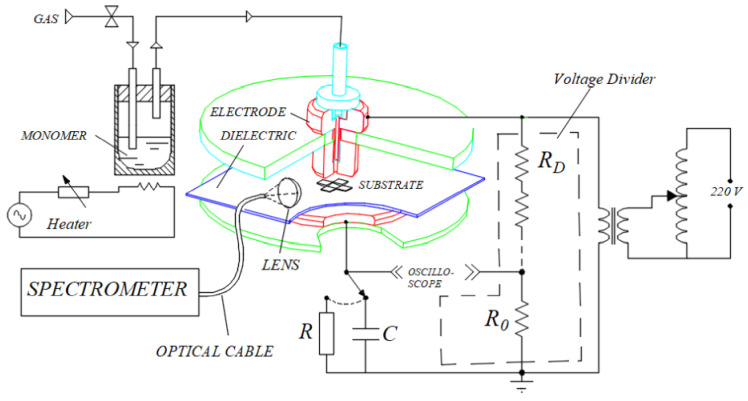
Schematic representation of the experimental setup used for film deposition and discharge characterization [36].

The plasmas used in this study were established using the system fully described elsewhere [36,37] and are shown schematically in Figure 1. It consists of a brass cylinder (2 cm in diameter) and an aluminum disk (5.6 cm in diameter) electrodes fitted in parallel, 3 mm apart from each other, in PTFE discs. The upper brass electrode was connected to an adjustable high voltage transformer (up to 20 kV peak-to-peak, 60 Hz) while the grounded lower disc was covered by a polyethylene sheet and served as a sample holder. Argon (99.9% pure), used as a carrier gas and to help in plasma ignition, was fed through a temperature-controlled stainless steel vessel containing Eugenol (4-Allyl-2-methoxyphenol, at least 98% purity from Sigma-Aldrich). This mixture, whose flow was controlled by rotameters, was injected through an axial hole at the center of the upper electrode. The discharge area was protected from the environment by a 40 mm diameter transparent polyethylene terephthalate hollow cylinder. The peak applied voltage *v_A_* was measured with a voltage divider while discharge current and charge were determined to measure the voltage drops across a resistor *R* (57 Ω) and a capacitor *C* (10 nF), respectively, connected in series with the ground electrode. The signals were recorded using a 500 MS/s digital storage oscilloscope (Tektronix TDS1001C-30EDU, Tektronix China Co. Ltd., Shangai, China) to calculate the mean discharge power P, for applied voltage much larger than in the capacitor, given by Equation (1):

(1) $$P=\frac{1}{T}\int_{0}^{Q}v_{A}dq$$

where *Q* is the charge stored in the capacitor during one period *T* = 1/60 s^−1^ of the signal.

Chemical structure, aging, stability and degradation of the films were characterized by infrared reflectance absorbance spectroscopy (IRRAS), using a Jasco FTIR 410 (Fourier-transform infrared, Jasco Corp., Tokyo, Japan) spectrometer co-adding 128 scans with a resolution of 4 cm^−1^. Film thickness and roughness were measured, at least 10 times in different locations on each sample, with a surface profilometer (Veeco D150, Veeco Metrology, Tucson, AZ, USA). To evaluate the thickness, the films were deposited on stainless steel slides partially masked with Kapton tape and the height of the step formed after removing the tape was measured with the profilometer.

The optical emission spectra of argon plasmas in the presence and the absence of Eugenol was measured using an Ocean Optics spectrometer (USB4000, Ocean Insight, Rochester, NY, USA). The spectrometer was connected to a fiber optic and the light was collected from a collimating lens located 3 mm from the edge of the electrode as shown in Figure 1. Electron temperature, *T_e_*, was estimated using the Boltzmann plot method assuming local thermodynamic equilibrium [3,38] and using Equation (2):

(2) $$\ln\frac{I_{ji}\lambda_{ji}}{A_{ji}g_{j}}=-\frac{E_{j}}{kT_{e}}+\ln\frac{hcN_{b}}{4\pi Z(T)}$$

where *I_ji_* is the intensity and *λ_ji_* is the wavelength of the measured spectral line emitted in an electron transition from an exciting level with energy *E_j_* to a lower state with energy *E_i_*, *g_j_* is the statistical weight of the excited level, *A_ji_* is the probability of the transition between the two states responsible for the measured emission, *k* is the Boltzmann’s constant, *c* the speed of light, *h* is the Planck’s constant, *N_b_* the total species population, and *Z*(*T*) corresponds to the partition function at a temperature *T*.

The electronic temperature has been derived from the reciprocal of the slopes of plots of $\ln\frac{I_{ji}\lambda_{ji}}{A_{ji}g_{j}}$ versus *E_j_* using the data provided by the *NIST* atomic database (Table 1).

## 3. Results and Discussion

### 3.1. Electric Characteristics

#### 3.1.1. Voltage and Current Waveforms

**Figure 2:**
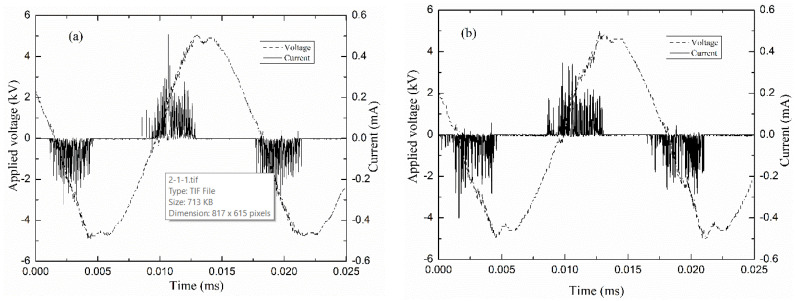
Typical current and voltage waveforms of discharge ignited at 10.0 kV peak-to-peak with 4 L/min total gas flow of (**a**) argon and (**b**) mixture of argon and Eugenol.

The electric characteristics of the DBD plasmas were evaluated by the measurement of the voltage drop [39,40,41,42] on the capacitor *C* and the resistor *R* defined in Figure 1. Figure 2 shows typical waveforms of applied voltage and current in argon discharges pure (a) and mixed with Eugenol (b). As can be observed, the discharge current curves contain several short-time spikes per each half cycle of the applied voltage, which indicates the filamentary regime of the discharges [42].

**Figure 3:**
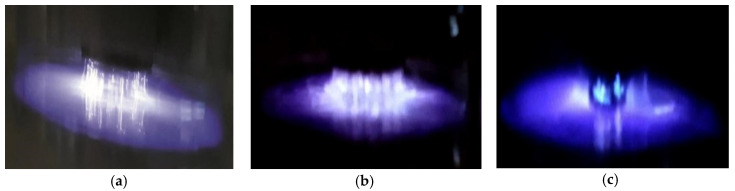
Pictures of discharges at 10.0 kV peak-to-peak and 4 L/min flow rate. (**a**) Pure argon; (**b**) mixture of argon and Eugenol; (**c**) mixture of argon and Eugenol with a stainless-steel substrate on the mylar covering the lower electrode.

The filamentary nature of the discharges can be better observed if one considers the pictures in Figure 3a,b which present photographs of discharges established under the same conditions as those used to obtain the curves in Figure 2a,b, respectively. As it can be observed, the filamentary discharges cover the entire surface of the electrode without any noticeable differences between the discharges in Ar or ArEu. However, a situation completely different is observed when the stainless-steel substrate is placed on the lower electrode. As one can see in Figure 3c, the introduction of the substrate causes the filaments to concentrate on the substrate. This is due to the distortion of electric field lines promoted by the sharp borders of the substrate. This result is similar to the previously reported helium DBD plasma [33,43]. In general, the discharge regime of the argon was not altered by the addition of the Eugenol monomer and works well for polymer deposition.

#### 3.1.2. Lissajous Figures and Plasma Power

**Figure 4:**
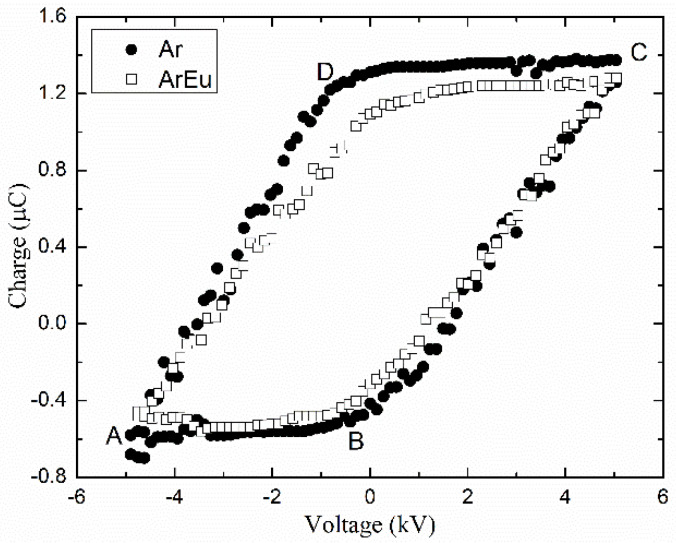
Lissajous figures of discharges were established in argon and argon with Eugenol (ArEu) at 10.0 kV peak–peak voltage and 4 L/min gas flow rate.

The power of the discharge was estimated by *Q*-V plots or the Lissajous figures of both pure argon and argon mixed to Eugenol (ArEu) plasmas as illustrated in Figure 4. In this picture, the slopes of lines DA and CB are related to the phase when the plasma is formed in the gap and provide an approximate value of the effective capacitance [44]. According to Equation (1), the mean power in one cycle can be calculated from the area of the Lissajous figure [37]. It can be seen in Figure 4 that the addition of Eugenol also reduces the mean discharge power. This is likely due to the reduction of the plasma current as electrons may be entrapped by Eu molecules.

**Figure 5:**
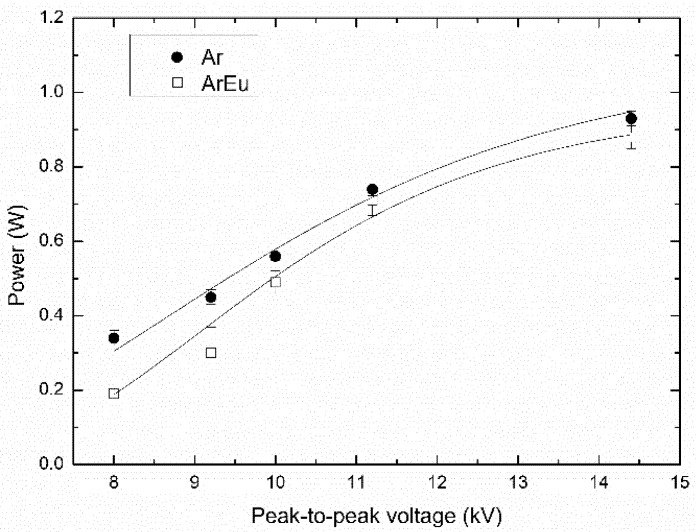
Mean power as a function of the applied voltage in Ar and Ar mixed with Eugenol at 4 L/min flow rate.

Figure 5 shows the variation of the power in plasmas established in Ar and ArEu as a function of the applied voltage. In both cases, the power increased as the voltage was increased, which is in agreement with previously reported works [37,45,46].

### 3.2. Optical Emission Spectroscopy of the Discharges

**Figure 6:**
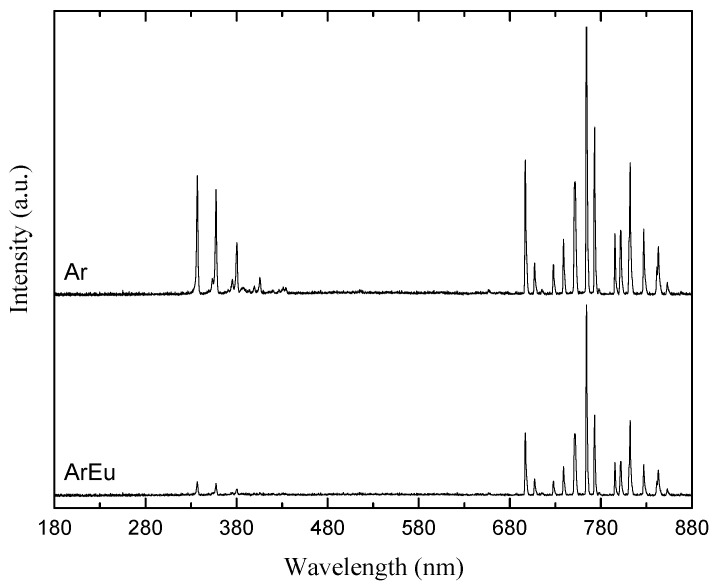
Emission spectra of dielectric barrier discharge DBD plasma in argon and mixture of argon with Eugenol (ArEu) measured at 10.0 kV peak-peak and 1 L/min flow rate.

Figure 6 shows typical optical emission spectra of plasmas ignited in Ar and ArEu at 10 kV peak-to-peak and 1 L/min flow rate. In the spectrum of Ar discharges there can be identified four emission lines ascribed to nitrogen molecules in the range 300–415 nm and several lines of argon (ArI) in 680–880 nm. In addition, there can be observed weak emission lines at 777 and 844 nm attributed to atomic oxygen (OI), generated via reactions of metastable Ar atoms or electrons with O_2_ in the surrounding air [47]. The presence of such lines due to the interaction of the plasma with the surrounding gas environment is also observed in helium DBD [3].

Additionally, the decrease in the intensity of almost all lines, especially N_2_ s positive lines, when Eu was added to the gas feed can be observed. This is associated with a decrease in plasma power, as observed in Figure 5, and the reduction in mean electron energy, and consequently, the concentration of the excited N_2_ species decreases. Although Eu molecules contain hydrogen, carbon, and oxygen atoms, it emission lines of species such as H, OH, and CH, indicating a low degree of monomer fragmentation were not detected. That indicates the preservation of the monomer rather than the degradation upon the plasma polymerization, which is in good agreement with the obtained Fourier-transform infrared (FTIR) spectra of polymeric films.

#### 3.2.1. Effect of Discharge Parameters on Plasma Composition

**Figure 7:**
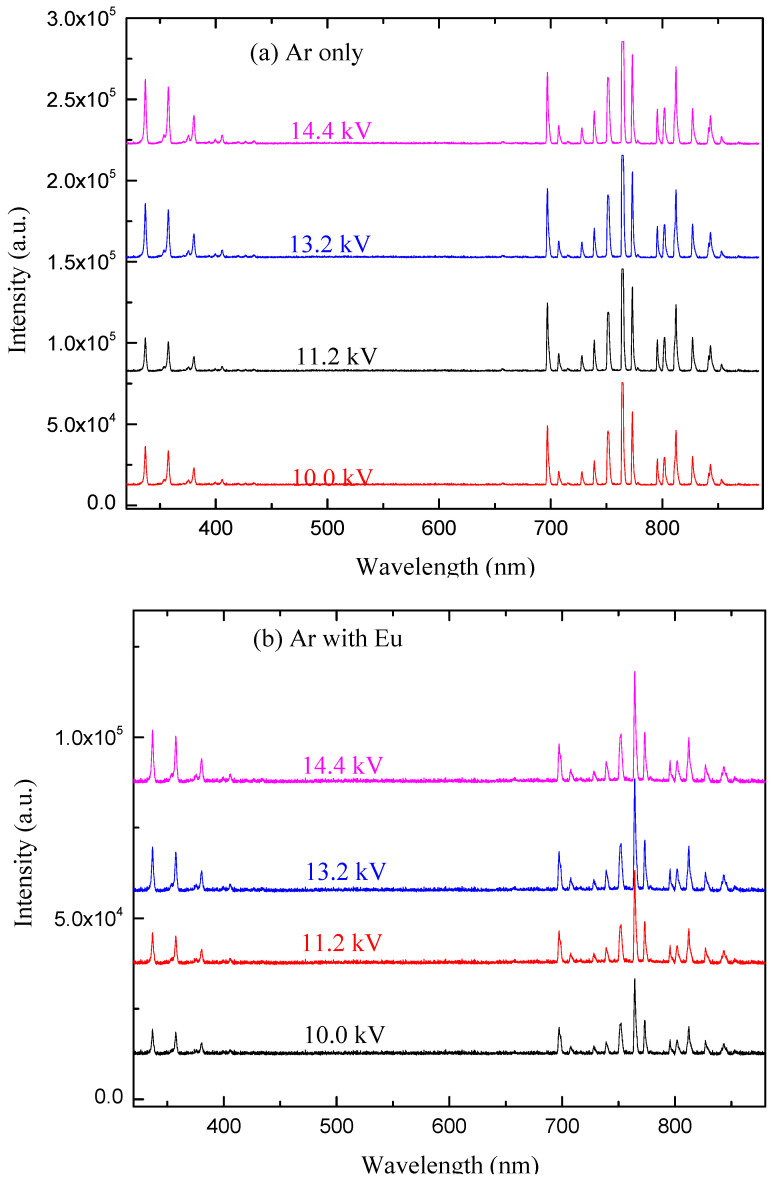

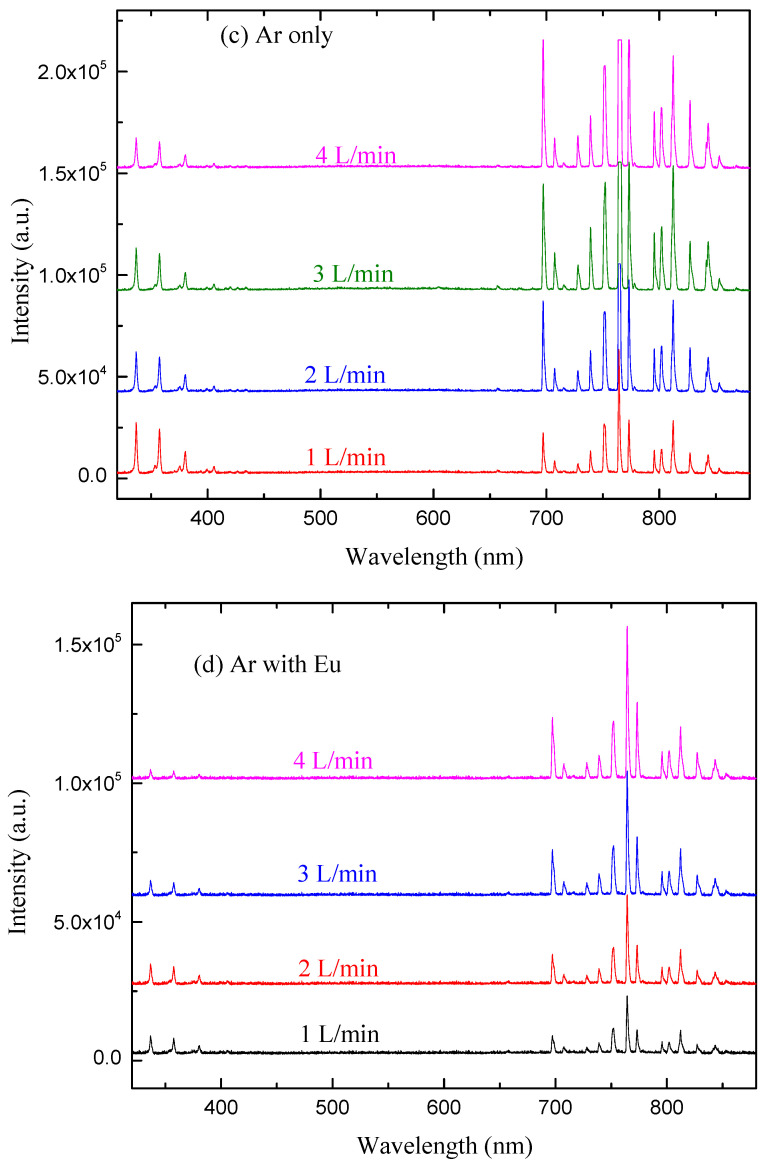
The optical emission lines of the argon only and argon with Eugenol discharge measured by varying, respectively, (**a**,**b**) peak-to-peak voltage at 1 L/min gas flow rate, (**c**,**d**) gas flow rate at 10.0 kV peak-peak voltage.

Figure 7 shows the effect of plasma discharge conditions, such as applied voltage and gas flow rate, on the optical emission line intensity for discharges of Ar and ArEu. According to this result, the intensities of the lines either increases monotonically or diminishes depending on the discharge condition, whereas the wavelength does not change. When we see these effects individually, the line intensity increases with voltage as shown in Figure 7a,b. This is due to the fact that the free electrons gain more energy due to the enhancement of the electric field in the gap and consequently, they increase the concentration of the excited neutral atoms and molecules. That is in agreement with the previously reported argon emission line of DBD plasma [48]. Similarly, the intensity of ArI lines in the region of 680–880 nm increases with the gas flow rate, while the emission line of N_2_ decreases as shown in Figure 7c,d. The possible explanation is that at a high Ar flow rate there is a reduction in contaminant species, namely N_2_, causing the rise of the density of Ar in detriment to N_2_.

**Figure 8:**
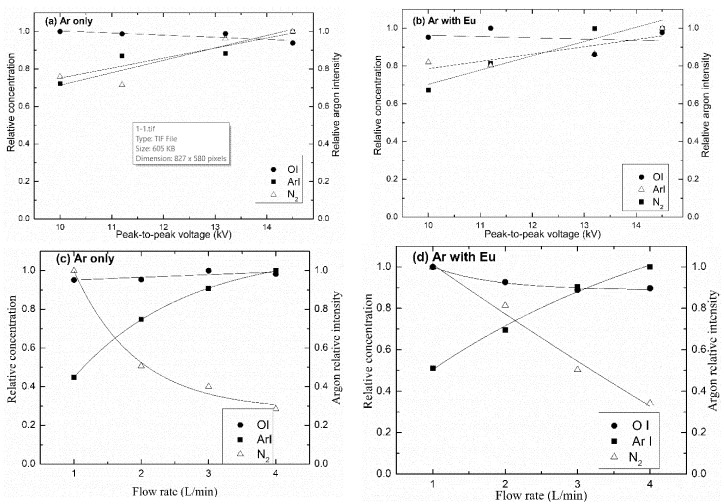
Variation of the relative concentration of N_2_, ArI, and OI for line intensity signals of (337 and 357 nm) N_2_, (750 and 811 nm) ArI, and 844 nm OI with, (**a**,**b**) peak-to-peak voltage at 1 L/min gas flow rate, and (**c**,**d**) gas flow rate at 10.0 kV peak-to-peak voltage.

The influence of applied voltage and gas flow rate on the relative concentration of N_2_, ArI, and OI for line intensities of the discharge in both Ar and ArEu was also investigated. Using the 811 nm Ar line as a reference, the concentration of those respective species in the plasma increase with the applied voltage, at 337 and 357 nm for N_2_, at 750 and 811 nm for ArI, and at 844 nm for OI, as can be seen in Figure 8a,b. This is due to the presence of energetic electrons associated with the enhancement of the electric field in the gap. As a consequence, there is an increase in the relative concentration/intensity of the excited neutral atoms and molecules [40,49]. That is in agreement with the previously reported argon emission line of DBD plasma [48,50]. Figure 8 also shows the relative argon intensity, which is indicative of plasma activity, that is, its ability to promote atoms to excited state, and nitrogen relative concentration in the plasma.

Similarly, the relative concentration of ArI and OI lines increases with the gas flow rate, while the N_2_ line decreases, as can be seen in Figure 8c,d, as illustrated. The possible explanation is that at a high Ar flow rate there is a reduction in contaminant species, namely N_2_, causing the rise of the density of Ar in detriment to N_2_. It is also noticed that the concentration of N_2_ decreases significantly with the flow rate for ArEu discharge compared to only Ar discharge. This is related to a more effective energy transfer of electrons to the monomer due to the high reactivity of its vinyl group in their chemical structure [50], which is in agreement with the decreasing effective discharge power (Figure 5).

#### 3.2.2. Effect of Discharge Parameters on Electron Temperature

**Figure 9:**
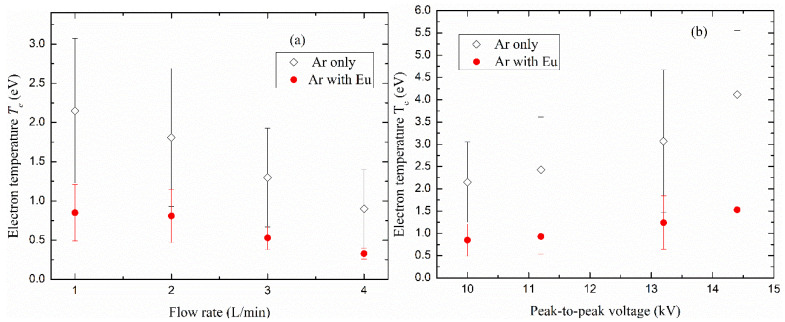
Relationship of electron temperature of the DBD plasma of argon and an admixture of argon with Eugenol discharge with (**a**) gas flow rate at 10 kV peak-to-peak voltage, (**b**) peak-to-peak applied voltage at 1 L/min flow rate.

The variation of electron temperatures was estimated in this work using the Boltzmann plot method, according to Equation (2) under Boltzmann approximation, using the neutral argon emission lines listed in Table 1, where *E_j_* and *E_i_* are the energies of the upper and lower levels of the radiative transitions, respectively. Figure 9 shows the effect of gas flow rate and applied voltage on electron temperature *T_e_* for Ar and ArEu DBD plasma. It is clear in Figure 9a that *T_e_* decreases from 2.2 ± 0.9 to 0.9 ± 0.5 eV and 0.9 ± 0.4 to 0.4 ± 0.1 eV when the gas flow rate increases from 1 to 4 L/min for Ar and ArEu, respectively. This fact may be explained as follows. This is due to the fact that the rise of gas density in the discharge region with flow rate leads to the reduction of the mean energy of the electrons due to the augment of collisions with other particles [51]. On the other hand, the rise of flow rate decreases the Yasuda factor given by the relation *W*/(*FM*), where *W* is the power input, *F* is the flow rate and *M* is the molecular weight of the monomer. A low value of this factor corresponds to the lower fragmentation rate of the monomer leading to a higher chemical structure similarity of the film with the monomer [52,53]. It is in good agreement with our FTIR spectra that show preservation of the spectrum of the monomer to the films. This dependence of the electron temperature with flow rate is also observed in the DBD discharge of argon with methane plasma [46].

On the other hand, *T_e_* of both Ar and ArEu rises with the applied voltage. The enhancement of the electric field increases the temperature from 2.2 ± 0.9 to 4.1 ± 1.4 eV and 0.9 ± 0.3 to 1.5 ± 0.7 eV, respectively, when a peak-to-peak voltage increases from 10.0 to 14.4 kV at a 1 L/min gas flow rate, as shown in Figure 9b. This enhancement of the electron temperature due to the electric field has been reported in other similar experiments [43,48], which indicates the field as the major energy source. Finally, when we compare *T_e_* of Ar and ArEu discharges, we observe that *T_e_* decreases significantly with the addition of monomer as a consequence of the increase in the collision frequency.

### 3.3. Thickness and Roughness of the Film

**Figure 10:**
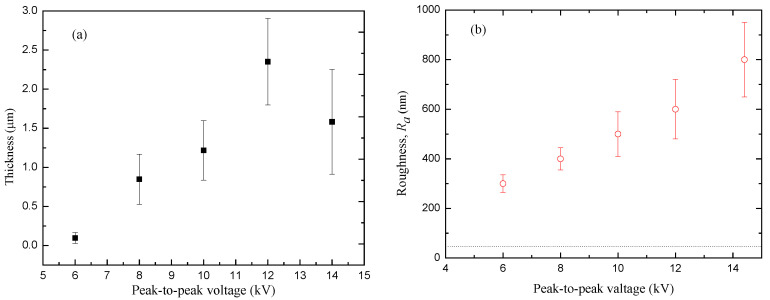
Variation of (**a**) thickness, (**b**) roughness of polyEugenol thin film with applied voltage for a 30 min deposition time, and a 4 L/min gas in a flow rate.

Plasma polymeric thin films were successfully deposited from the Eugenol monomer on a stainless steel slide under a different applied voltage. As mentioned in the Introduction, several plasma parameters can influence the properties of the deposited thin films. Figure 10a shows the variation of film thickness *h* with a peak-to-peak voltage at 4 L/min gas flow rate and 30 min deposition time. The thickness of the film increases from 1.0 to 2.4 µm, with the applied voltage from 6.0 to 12.0 kV. After that, it declines to about 1.6 µm for further increases in the voltage up to 14.0 kV. The rising of the electric field enhances the fragmentation process and the concentration of active species involved in plasma polymerization reactions. As a consequence, the film becomes thicker in proportion to the field intensity. However, over a certain intensity, two competing factors occur in the polymerization process, the increase in the substrate temperature and the plasma ablation, which leads to the reduction of the film thickness. The films at higher field intensity are thinner and denser with a high degree of cross-linkage [36,54]. It should be also observed that the substrate temperature influences the adsorption of non-excited and fragmented molecules or slightly fragmented precursors during the film growing process. Such rising and decaying of the film thickness with the applied voltage are in good agreement with the absorption intensity of the functional groups shown by the FTIR spectrum [36,54].

The roughness *R_a_* of the films as a function of the applied voltage is shown in Figure 10b. For the bare steel, the *R_a_* value is represented by the dotted line. It clearly shows that the roughness of the film increases with applied voltage as do the uncertainty values. This behavior on the roughness and its uncertainty are proportional to the number of valleys produced by the plasma filaments in the film, which rises with the applied voltage. On the other hand, according to the scaling law, as the film thickness increases, the level of grain size and roughness also increases, until saturation [55]. This saturation occurs at high voltage with a drop in the thickness and still rises of roughness by field effect.

### 3.4. Molecular Structure

**Figure 11:**
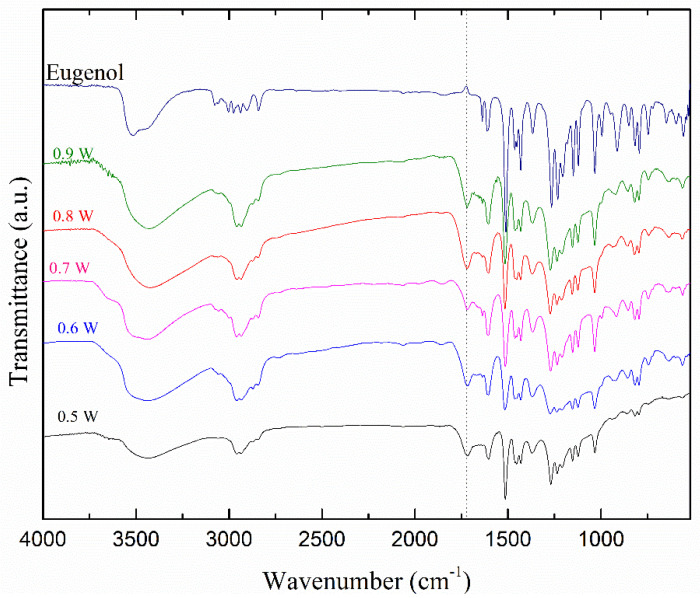
FTIR spectra of Eugenol and polyEugenol thin films deposited with a 30 min deposition time and a 4 L/min gas flow rate by varying the plasma discharge power from 0.5 to 0.9 W.

To complete the structural study of the polymeric thin film, FTIR analysis of the monomer (Eugenol) was also carried out in addition to the film deposited under different plasma discharge conditions as shown in Figure 11.

Considering first the spectrum for the monomer Eugenol, a broad peak centered at 3519 cm^−1^ corresponds to O–H stretch, indicating the presence of phenol. The absorption bands at 1234 and 1030 cm^−1^ are assigned to the stretching vibrations of C–O of the phenyl-alkyl ether (methoxy group) and phenol, respectively. The weak peaks in the range of 649–555 cm^−1^ are attributed to the C–O in-plane bending of methoxy and the peak at 1209 cm^−1^ to the O–H in-plane deformation of phenol. The peaks at 1450 cm^−1^ are attributed to the asymmetric bending vibration of C–H in the methoxy group and the peak at 1363 cm^−1^ to the symmetric one. The asymmetric stretching vibration of C–H in the methoxy group of phenyl alkyl ethers is also observed in the region of 2973–2940 cm^−1^ and its corresponding symmetric one is observed at 2841 cm^−1^. In the same wavelength region, a peak at 2903 cm^−1^ indicates the C–H vibrational stretching of the CH_2_=C–H (vinyl) group. Additionally, two absorption bands at 995 and 916 cm^−1^ are attributed to the C–H out-of-plane bending vibration of the vinyl group. In the high wavenumber region, the weak peaks appeared at 3060 cm^−1^ and 3004 cm^−1^ which corresponds, respectively, to the C–H stretching vibrational of aromatic skeletal and vinyl alkene group. Moreover, the weak peaks between 848 and 746 cm^−1^ are attributed to the C–H out-of-plane deformation of the 1, 2, and 4-tri-substituted benzenoid compounds.

The FTIR spectra of the monomer and the films are quite similar, except for the reduction of peak intensity in the film, particularly in the fingerprint region below 1500 cm^−1^. This reduction means that a small quantity of monomer was polymerized and deposited. Additionally, the peaks at 2973 and 2903 cm^−1^ are assigned to asymmetric and symmetric stretching of methylene and are present only in the monomer spectra due to the loss of the vinyl group. It is interesting to point out that the vinyl double bond was broken during the plasma polymerization processes, just like conventional polymerization. Moreover, the peak at 3519 cm^−1^ shifts towards a lower frequency at 3432 cm^−1^. That might be associated with the formation of intermolecular H-bonding caused by plasma polymer formation. On the other hand, a peak at 1705 cm^−1^ associated with ketone appeared in the film, which is absent in the spectrum of the monomer. That corresponds to the post oxidation of the trapped free radicals confined during the formation of the film and the tautomerization as well [56,57].

Figure 11 also gives the spectra of films deposited for various discharge power up to 0.9 W, which corresponds to applied voltage up to 14.4 kV peak-to-peak. As can be seen, all functional groups are preserved in the film and the intensity of absorption peaks increases with the power of discharge. The partial oxidation of the film, particularly for –OH and aryl groups, leads to a slight decrease in the intensity and the bandwidth for discharges with power above 0.7 W. It is interesting to mention the preservation monomer functional groups, particularly hydroxy and aromatics that are responsible for antimicrobial activity.

### 3.5. Stability and Aging

Several medical devices are often require storage for a certain period before they are used [58]. Therefore, films produced for biomedical applications require stability in atmospheric air for extended periods and in aqueous solution as well. Moreover, good adhesion of the films to the substrate and the preservation of its functionality after polymerization is critical in these applications. Since the initial attachment of bacteria assay was carried out in the TSB solution, it is worth seeing the physical and chemical stability of film in this medium [59].

**Figure 12:**
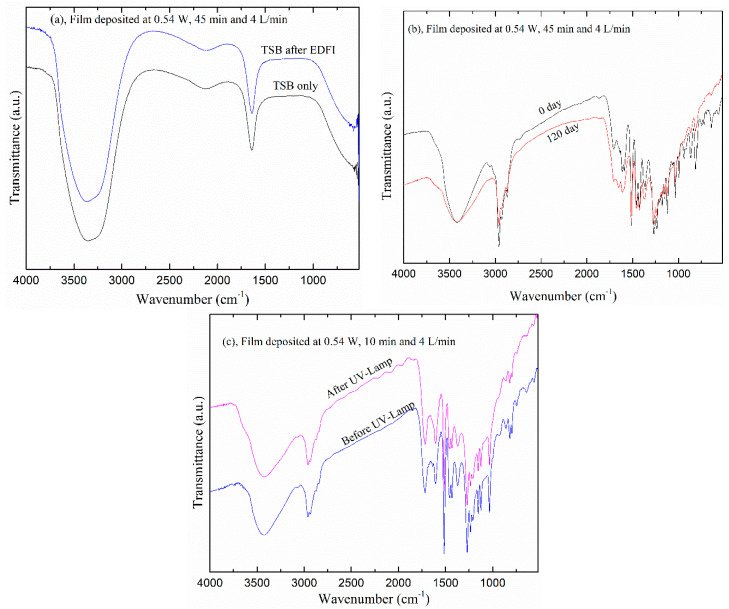
The FTIR spectra of (**a**) Tryptic Soy Broth (TSB), and of the film exposed to (**b**) ambient air for 120 days and (**c**) to UV radiation for 1 h. EDFI is eugenol-derived film immersion.

In order to investigate these features the structure of Eugenol-derived films was investigated with FTIR for attacks in Tryptone Soy Broth (TSB) solution, and by exposure to atmospheric air and a high thermal UV-lamp. The spectra of TSB before and after immersion of the films for 45 min are shown in Figure 12a. They have a close similarity which indicates good stability of the films on the surface of the substrate without any degradation. The spectra of the film after 120 days in ambient air are shown in Figure 12b. Again, there is no change in the obtained spectra, which shows that the obtained films are not affected by exposure to the ambient air for this period. For UV analysis, the films were exposed to a high thermal UV-lump (at 56 °C) at a nearby 3 cm distance for 1 h, which is a typical procedure for material sterilization. The resulting spectra are shown in Figure 12c indicating that the films are unaffected by the high energy radiation. Therefore, these tests showed that the Eu film produced in the DBD plasma is thermally stable with adhesion on a stainless steel substrate, maintaining its properties for a long period in a regular atmosphere.

## 4. Conclusions

In the present study, we deposited and characterized the Eugenol-derived film, diagnosed the dielectric barrier discharge, and estimated the electron temperature of argon plasma in the presence and the absence of monomer Eugenol, assuming local thermodynamic equilibrium. The electron temperature of the ArEU discharge was around 1.5 eV for gas flow rate 1 L/min and 14.4 kV peak-to-peak voltage. The filamentary discharge of the device was successfully used to polymerize PolyEugenol thin film on a stainless steel substrate under different discharge conditions. The obtained thin film had 2400 nm maximum thickness and 800 nm roughness with a molecular structure similar to the monomer. They are also thermally stable, undegraded, and unaged when exposed to a high thermal UV-lamp, TSB solution, and atmospheric air for long periods of time. These results encourage the use of polyEugenol thin film coatings, for instance, for suppression of biofilm formation and corrosion protection of biomaterials.

## Figures and Tables

**Table 1:** Spectroscopic Data for the Observed ArI Lines Used for the Evaluation of *T_e_*. All Lines Are Emitted During 4p-4s Transitions.

Wavelength (nm)	*E_i_* (eV)	*E_j_* (eV)	*A_ji_* × 10^7^ (s^−1^)	*g_j_*
696.54	11.54	13.32	0.63	3
738.39	11.62	13.30	0.84	5
763.50	11.54	13.17	2.45	5
772.37	11.54	13.15	0.51	3
794.82	11.72	13.28	1.86	3
811.53	11.54	13.07	3.31	7
826.45	11.82	13.32	1.54	3
